# A comparison of laboratory-based and office-based Framingham risk scores to predict 10-year risk of cardiovascular diseases: a population-based study

**DOI:** 10.1186/s12967-023-04568-8

**Published:** 2023-10-03

**Authors:** Azizallah Dehghan, Sajjad Ahmadnia Motlagh, Rozhan Khezri, Fatemeh Rezaei, Dagfinn Aune

**Affiliations:** 1 Noncommunicable Diseases Research Center, Fasa University of Medical Sciences, Fasa, Iran; 2 Student Research Committee, Jahrom University of Medical Sciences, Jahrom, Iran; 3 Department of Epidemiology, School of Public Health, Iran University of Medical Sciences, Tehran, Iran; 4 Research Center for Social Determinants of Health, Jahrom University of Medical Sciences, Jahrom, Iran; 5 Department of Epidemiology and Biostatistics, School of Public Health, Imperial College London, London, UK; 6 Department of Nutrition, Oslo New University College, Oslo, Norway

**Keywords:** Framingham risk score, Laboratory-based, Office-based, Cardiovascular disease, Risk prediction, Agreement, Correlation

## Abstract

**Background:**

Two versions of Framingham’s 10-year risk score are defined for cardiovascular diseases, namely laboratory-based and office-based models. The former is mainly employed in high-income countries, but unfortunately, it is not cost-effective or practical to utilize it in countries with poor facilities. Therefore, the present study aims to identify the agreement and correlation between laboratory-based and office-based Framingham models.

**Methods:**

Using laboratory-based and office-based Framingham models, this cross-sectional study used data from 8944 participants without a history of CVDs and stroke at baseline in the Fasa cohort study to predict the 10-year risk of CVDs. The laboratory-based model included age, sex, diabetes, smoking status, systolic blood pressure (SBP), treatment of hypertension, total cholesterol, and high-density lipoprotein (HDL); and the office-based model included age, sex, diabetes, smoking status, SBP, treatment of hypertension, and body mass index (BMI). The agreement between risk categories of laboratory-based and office-based Framingham models (low [< 10%], moderate [from 10 to < 20%], high [≥ 20%]) was assessed by kappa coefficients and percent agreement. Then, the correlation between the risk scores was estimated using correlation coefficients and illustrated using scatter plots. Finally, agreements, correlation coefficient, and scatter plots for laboratory-based and office-based Framingham models were analyzed by stratified Framingham risk score factors including sex, age, BMI categories, hypertension, smoking, and diabetes status.

**Results:**

The two models showed substantial agreement at 89.40% with a kappa coefficient of 0.75. The agreement was substantial in all men (kappa = 0.73) and women (kappa = 0.72), people aged < 60 years (kappa = 0.73) and aged ≥ 60 years (kappa = 0.69), smokers (kappa = 0.70) and non-smokers (kappa = 0.75), people with hypertension (kappa = 0.73) and without hypertension (kappa = 0.75), diabetics (kappa = 0.71) and non-diabetics (kappa = 0.75), people with normal BMI (kappa = 0.75) and people with overweight and obesity (kappa = 0.76). There was also a very strong positive correlation (r ≥ 0.92) between laboratory-based and office-based models in terms of age, sex, BMI, hypertension, smoking status and diabetes status.

**Conclusions:**

The current study showed that there was a substantial agreement between the office-based and laboratory-based models, and there was a very strong positive correlation between the risk scores in the entire population as well across subgroups. Although differences were observed in some subgroups, these differences were small and not clinically relevant. Therefore, office-based models are suitable in low-middle-income countries (LMICs) with limited laboratory resources and facilities because they are more convenient and accessible. However, the validity of the office-based model must be assessed in longitudinal studies in LMICs.

**Supplementary Information:**

The online version contains supplementary material available at 10.1186/s12967-023-04568-8.

## Introduction

Cardiovascular diseases (CVDs) account for 32% of all deaths and remain the leading cause of morbidity and mortality worldwide [1]. CVDs contribute to a substantial burden on healthcare systems in LMICs. It has been projected that 24 million incident CVD cases will occur by the end of 2030 [2, 3]. It has also been projected that the prevalence of CVD will increase to 130 million people by 2035 globally. In the United States, the total economic costs of CVD have been estimated at $1.1 trillion [4].

CVD is the leading cause of mortality in Iran and resulted in one million disability-adjusted life years (DALY) [5]. It is estimated that DALYs related to CVD will double between 2005 and 2025 among Iranian adults aged ≥ 30 years [6].

The World Health Organization (WHO) asserts that it is possible to prevent 75% of premature CVDs [3, 7] by measuring and intervening on some of its main risk factors such as unhealthy diet, inactivity, smoking, and alcohol consumption, risk factors which can lead to hypertension, high blood sugar (hyperglycemia), high cholesterol (hyperlipidemia) and overweight and obesity. Primary prevention and early detection could also reduce the economic burden of CVDs on the healthcare systems [8].

If people at high risk of CVDs, especially those at risk of nonfatal myocardial infarction, stroke, or CVD death could be identified timely and accurately through assessment of various risk factors, various management tools provide an opportunity to modify the risk factors and minimize the likelihood of CVD occurrence [9]. These tools are likely to help people increase their awareness, modify their lifestyles and reduce their mortality [10]. Developed by D’Agostino et al. in 2008, the Framingham Risk Score (FRS) is considered one of the first and most well-known risk scores with laboratory-based and office-based models to predict 10-year risk of cardiovascular diseases [9]. The laboratory-based model calculates risk scores based on sex-specific levels of risk factors such as age, diabetes, smoking status, SBP, treatment of hypertension, and HDL cholesterol while the office-based model is similar, but replaces measurement of total cholesterol and HDL cholesterol with measurement of BMI [11].

Several studies have shown that Framingham risk scores with laboratory-based and office-based models can be applicable in different regions around the world [12–17]. The predictive power of these models was shown in a cohort study with long-term follow-up [12], and have also been shown to be high in several other studies [13–17]. In Iran, several studies used the Framingham risk score [18, 19]. Mirzaei et al. used the office-based Framingham risk score and showed that 30.6%, 42.2%, and 26.5% were in low-, moderate-, and high-risk groups [18]. But few studies have compared the laboratory-based and office-based Framingham risk scores. In 2021, Rezaei et al. showed that there is good agreement between laboratory-based and non-laboratory-based Framingham models in the Pars cohort study [17]. We further investigated these associations in the Fasa cohort study, a cohort of different ethnic groups including Turk, Fars, and Arab, which have differences in lifestyle and socio-economic status compared to other ethnic groups. Also, all analyses in this study were done based on all strata of cardiovascular risk factors separately.

It is less likely that laboratory-based models can be used in LMICs due to lack of or poor laboratory infrastructure, limited resources, lack of or insufficiently trained staff. Office-based models could be substituted for laboratory-based models to determine the risk score, if shown to have similar predictive value. There was a strong correlation (78%) between cholesterol-based and BMI-based Framingham models in a US study [20]; however, the main question raised here is whether there is an agreement between laboratory-based and office-based Framingham models and whether an office-based model can be substituted for a laboratory-based model or not. Therefore, the present study aims to determine the agreement and correlation between laboratory-based and office-based Framingham risk models.

## Methods

This cross-sectional study was carried out based on baseline data from the Fasa cohort study in the southern Fars province in southern Iran, and details of the study have been published previously [21]. The Fasa Cohort Study included a total of 10,138 participants aged > 35 living in Shesh Deh and Qarah Bulaq District, in Fasa County. The Fasa cohort study is a part of the Prospective Epidemiological Research Studies in IrAN (PERSIAN) cohort. The PERSIAN cohort study aims to evaluate risk factors for non-communicable diseases. More details about PERSIAN cohorts and the Fasa cohort study (protocol, laboratory measurements, and physical examinations) have been published previously [21–23].

**Fig. 1:**
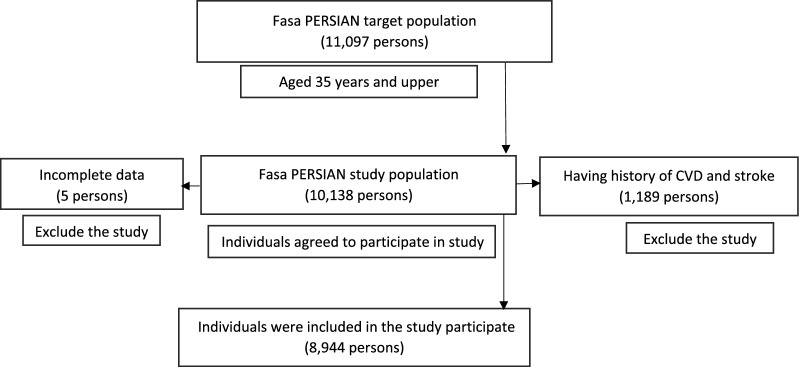
Flowchart displaying the sample selection process

In Fasa cohort study, the professional staff was in charge of collecting and recording the following data for each participant (10,138 participants in total): demographic data (age, gender, marital status, etc.), anthropometric characteristics (height and weight), and medical characteristics (illness history). A healthcare professional and a medical practitioner also conducted a brief physical examination and examined participants’ blood pressure (BP), blood sugar and lipids (total cholesterol, triglycerides, HDL, and LDL cholesterol). In this study, 1189 persons who had a history of CVDs and stroke at baseline were excluded. Finally, 8944 persons were included (Fig. 1).

### CVD risk score

The FRS equation was developed by D’Agostino et al. in 2008 [11]. The FRS is a sex-specific algorithm used to calculate the risk of developing CVD over the next 10 years. There are two methods for estimated CVD risk score; point-based and Cox-based CVD risk prediction models [11]. In this study, Cox-based CVD risk prediction model were used for calculation laboratory-based and office-based models.

Laboratory-based and office-based Framingham risk models were employed to predict the 10-year risk of CVDs. The laboratory-based models includes age, diabetes, smoking status, SBP, treatment of hypertension, total cholesterol, and HDL cholesterol and office-based models includes age, diabetes, smoking status, SBP, treatment of hypertension and BMI, with the difference being the former using total cholesterol and HDL cholesterol and the latter using BMI instead [11].

One study focused on the agreement between laboratory-based and office-based Globorisk scores and explained how the above-mentioned variables are measured [24]. To include participants in the study, the following experiments were conducted. First, the level of fasting blood sugar (10–14 h fasting), HDL, and total cholesterol were measured by laboratory tests. Second, the participants were interviewed about their smoking status. Third, prevalent diabetes status was defined as participants having a history of diabetes or abnormal fasting blood sugar ( ≥ 126 mg/L) at baseline. Fourth, BP was measured twice with a 15-min resting time and then its average was recorded. Finally, BMI was calculated as the weight in kilograms divided by the square of the height in meters, and categorized into two groups, normal weight (BMI < 25 kg/m^2^) and overweight and obese (BMI ≥ 25 kg/m^2^) participants.

### Ethical considerations

This study was approved by the Ethics Committee of Jahrom University of Medical Sciences (IR.JUMS.REC.1401.093). The data were collected anonymously and each participant informed consent forms.

### Statistical analysis

Quantitative variables such as numbers, percentages, means and standard deviations were calculated. A t-test and a chi-square test was used to determine if there is a statistically significant difference between the means of the variables.

In this study, the laboratory-based model was assumed to be the gold standard. Then one-sample t-test was done to compare the mean predicted risk of office-based and the laboratory-based CVD risk scores. Kappa coefficients and percent agreement were used to assess the relationship between the risk categories of the laboratory-based and office-based Framingham models and the results were divided in three categories, namely low (< 10%), moderate (10% to < 20%), and high (≥ 20%). We used the cut-offs reported by Venkatesh et al. to classify kappa coefficients into six groups: poor (≤ 0), slight (0.01–0.20), fair (0.21–0.40), moderate (0.41–0.60), substantial (0.61–0.80), and almost perfect (0.81–1.0) [25].

A scatterplot and correlation coefficients showed the correlation between two quantitative variables of laboratory-based and office-based models. The correlation coefficients range from − 1 to 1. The two extremes indicate a perfect correlation while zero means there is no linear correlation between variables. Thus, in this study, the correlation coefficients of 0.00–0.10, 0.10–0.39, 0.40–0.69, 0.70–0.89, and 0.90–1.0 indicated a negligible correlation between the two variables, weak correlation, moderate correlation, strong correlation, and very strong correlation, respectively [26]. One-sample t-test, kappa coefficients, correlation coefficients, and scatterplots were represented by stratified FRS factors including age, sex, BMI category, hypertension, smoking and diabetes status.

Statistical analyses of individuals demographics and CVD risk score were performed using the Statistical Package for Social Science (IBM SPSS Statistics for Windows, Version 23.0. Armonk, NY: IBM Corp). A p-value < 0.05 was considered statistically significant.

## Results

The participants had the following characteristics. Of 8944 participants without a history of CVDs and stroke at baseline and with a mean age of 47.72 ± 9.25 years were included in the analysis. Regarding the mean of the age group, 53.80% of participants were women with a mean age of 47.59 ± 9.14 years and 46.20% of participants were men with a mean age of 47.86 ± 9.37 years. The study population characteristics and prevalence of FRS factors are shown in Additional file 1: Table S1.

Table 1 illustrates the laboratory-based and office-based models mean risk scores in terms of age groups, sex, smoking status, diabetes, hypertension, and BMI. The laboratory-based model was considered as the gold standard for CVD risk assessment. The mean predicted risk of the office-based and laboratory-based models was compared by one-sample t-test. In general, the mean score of the office-based-model was higher than the laboratory-based-model (8.52 ± 9.67 vs. 8.30 ± 9.33) and this difference was significant (p < 0.05). The result showed that there is significant difference between the mean predicted risk of the office-base model and the mean predicted risk of the laboratory-based model in people aged 60 years and over, females, smokers and non-smokers, people with hypertension, diabetics, and those have overweight and obesity (p < 0.05).

**Table 1:** The mean of laboratory-based and office-based Framingham risk scores by stratified risk factors

Variables	Laboratory-based CVD risk score^a^ (10- year, %)	Office-based CVD risk score (10- year, %)	p-value
	Mean ± SD	Mean ± SD	
Age group			
< 60	6.60 ± 7.19	6.65 ± 7.09	0.53
≥ 60	19.33 ± 13.31	20.69 ± 14.28	0.001
Gender			
Male	12.54 ± 10.98	12.77 ± 11.25	0.19
Female	4.66 ± 5.44	4.88 ± 6.04	0.01
Smoking (now)			
No	6.77 ± 7.84	7.03 ± 8.35	0.007
Yes	14.51 ± 11.99	14.60 ± 12.04	< 0.001
Hypertension			
No	7.07 ± 7.64	7.11 ± 7.62	0.63
Yes	15.01 ± 13.79	16.21 ± 14.73	0.002
Diabetes			
No	7.45 ± 8.25	7.55 ± 8.42	0.29
Yes	15.30 ± 13.79	16.62 ± 14.44	0.005
BMI category			
Normal	8.40 ± 9.03	8.29 ± 8.84	0.40
Overweight and obesity	8.20 ± 9.59	8.74 ± 10.36	< 0.001
Total	8.30 ± 9.33	8.52 ± 9.67	0.03

^a^The laboratory-based model was considered as the gold standard

**Fig. 2:**
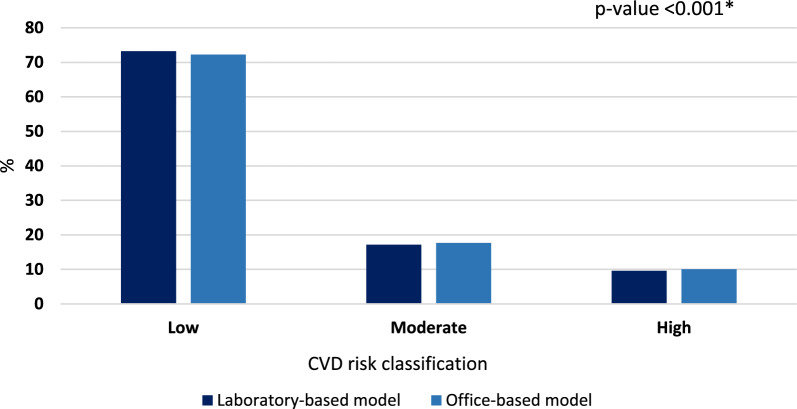
Percentage of the CVD risk classification of laboratory-based and office-based Framingham models. *p-value for kappa coefficient

The risk classification of laboratory-based and office-based models showed very similar distributions in the population, with 73.22% being low, 17.19% moderate and 9.59% high with the laboratory-based model and 72.28% being low, 17.68% moderate and 10.04% high with the office-based model (Fig. 2). Kappa coefficient showed the agreement between the two models was statistically significant (P < 0.001).

### The agreement between laboratory-based and office-based models by stratified risk factors

In the entire population, the agreement between laboratory-based and office-based models was substantial with a kappa coefficient of 0.75. The agreement was also measured in different subgroups in which there was almost perfect agreement in men, non-smokers, non-hypertensive subjects, non-diabetics, persons with normal BMI and those have overweight and obesity which was in the low-risk category. The agreement was moderate among men, smokers, diabetics and person that have hypertension which was in the moderate risk category (Table 2).

**Table 2:** Agreement between the laboratory-based and office-based models by stratified risk factor

Laboratory-based risk category	Office-based risk category			Percent agreement (kappa) in subgroups^a^	Percent agreement (kappa)^b^
	Low	Moderate	High		
Gender					
Male					
Low	2072	209	3	90.44 (0.81)	84.12 (0.73)
Moderate	183	796	137	84.20 (0.60)	
High	0	124	608	93.61 (0.78)	
Female					
Low	4131	133	1	95.57 (0.79)	93.93 (0.72)
Moderate	79	291	51	93.95 (0.63)	
High	0	28	98	98.33 (0.70)	
Age groups					
< 60					
Low	5928	280	4	93.38 (0.80)	90.89 (0.73)
Moderate	229	831	98	90.91 (0.65)	
High	0	95	286	97.45 (0.73)	
≥ 60					
Low	275	62	0	92.03 (0.80)	0.79.15 (0.69)
Moderate	33	256	90	97.45 (0.73)	
High	0	57	420	87.67 (0.75)	
Smoking status					
Smoker					
Low	715	112	2	87.95 (0.76)	81.08 (0.70)
Moderate	98	394	61	81.19 (0.56)	
High	0	60	318	93.01 (0.79)	
Nonsmoker					
Low	5488	230	2	94.40 (0.83)	91.43 (0.75)
Moderate	164	693	127	91.49 (0.64)	
High	0	92	388	96.92 (0.76)	
Hypertension					
Yes					
Low	584	92	2	90.11 (0.80)	82.61 (0.73)
Moderate	43	247	73	82.75 (0.56)	
High	0	31	314	92.35 (0.80)	
No					
Low	5619	250	2	93.76 (0.82)	90.64 (0.75)
Moderate	219	840	115	90.67 (0.65)	
High	0	121	392	96.85 (0.75)	
Diabetic status					
Diabetic					
Low	376	77	1	89.06 (0.78)	81.14 (0.71)
Moderate	27	182	151	81.25 (0.54)	
High	0	25	221	91.98 (0.80)	
Non-diabetic					
Low	5827	265	3	93.69 (0.83)	90.39 (0.75)
Moderate	235	905	137	90.43 (0.65)	
High	0	127	485	96.65 (0.77)	
BMI category					
Normal					
Low	2906	148	2	92.75 (0.82)	88.98 (0.75)
Moderate	157	557	69	89.02 (0.64)	
High	0	91	307	96.17 (0.77)	
Overweight and obesity					
Low	3297	194	2	93.60 (0.84)	89.78 (0.76)
Moderate	105	530	119	89.82 (0.63)	
High	0	61	399	96.20 (0.79)	
Total					
Low	6203	342	4	93.20 (0.83)	89.40 (0.75)
Moderate	262	1087	188	89.44 (0.63)	
High	0	152	706	96.15 (0.78)	

^a^Agreement (Kappa) between two models in subgroups

^b^Total agreement (Kappa) between two models

### Correlation coefficients between laboratory-based and office-based models by stratified risk factors

**Fig. 3:**
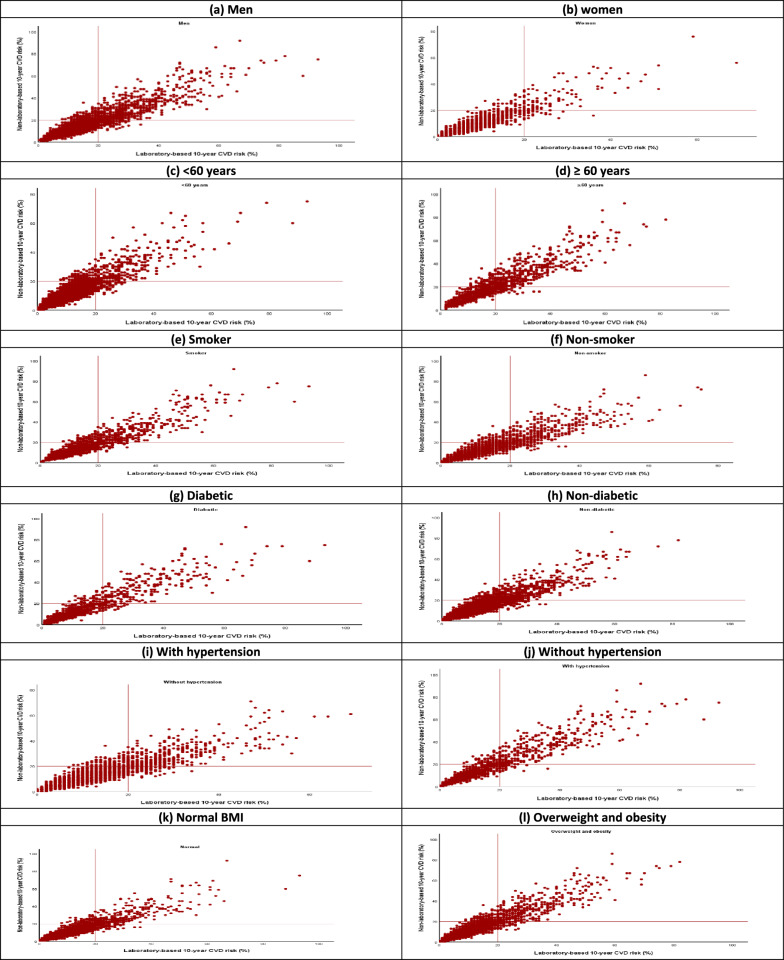
Scatter plot showing a linear relationship between Framingham laboratory-based and office-based models by stratified risk factors. **a** Scatter plot for men; **b** Scatter plot for women; **c** Scatter plot for < 60 years; **d** Scatter plot for ≥ 60 years; **e** Scatter plot for smoker; **f** Scatter plot for non-smoker; **g** Scatter plot for diabetic; **h** Scatter plot for non-diabetic; **i** Scatter plot for with hypertension; **j** Scatter plot for without hypertension; **k** Scatter plot for normal BMI; **l** Scatter plot for overweight and obesity

Table 3 illustrates the correlation between laboratory-based and office-based Framingham risk scores by stratified risk factors. There was a very strong positive correlation between laboratory-based and office-based models overall and across various subgroups (r = 0.92–0.95, p < 0.001) including age, sex, BMI, smoking status, diabetes, hypertension. The correlation between laboratory-based and office-based CVDs individual-level risk scores across subgroups is illustrated in the form of scatterplots (Fig. 3).

**Table 3:** Pearson correlation coefficient and corresponding p-values for laboratory-based and office-based Framingham risk scores by stratified risk factors

Variables	N	Correlation coefficient (r)	p-value
Gender			
Male	4132	0.94	< 0.001
Female	4812	0.94	< 0.001
Age group			
< 60	7751	0.94	< 0.001
≥ 60	1193	0.92	< 0.001
Smoking status			
Smoker	1760	0.94	< 0.001
Non-smoker	7184	0.95	< 0.001
Hypertension status			
Yes	1388	0.94	< 0.001
No	7558	0.94	< 0.001
Diabetic status			
Yes	960	0.93	< 0.001
No	7984	0.95	< 0.001
BMI category			
Normal	4237	0.94	< 0.001
Overweight and obesity	4707	0.95	< 0.001
Total	8944	0.95	< 0.001

## Discussion

This study found high agreement between and very strong correlations between laboratory-based and office-based Framingham Risk Score models for estimating risk of CVD, and this was also observed in strata of various other risk factors including age, sex, diabetes, hypertension, smoking status and obesity.

Our findings are consistent with a study in South Asia, which showed good agreement between laboratory-based and office-based models when stratified by various risk groups [27]. It is also consistent with Wekesah et al’s study which showed that the high-risk group is very close to each other in both models [28]. Another study also showed similar results for laboratory-based and office-based WHO models when stratified by various risk groups [29].

In the current study, concordance between risk categories of two models was calculated according to stratified FRS factors and agreement between two models in was almost perfect in the low-risk group and substantial in the moderate- and high-risk groups.

More men were in the high-risk group in both models. The mean risk scores of the office-based model were slightly higher than the laboratory-based model in men and women. This difference in women was significant, although not clinically significant. Other similar studies have also been conducted to evaluate the agreement between different CVD risk prediction models. For example, Rezaei et al. found the mean risk of CVDs in the office-based model was higher than in the laboratory-based model [17]. Guzman-Vilca et al. realized the similarity between the mean risk score of the WHO laboratory-based and office-based models [30]. The present study found that the mean risk scores of CVDs in men were about three times as high as those in women. This is consistent with a study by Guzman-Vilca et al., which showed higher mean risk scores in men than in women [30]. Borhanuddin et al. studied more than 53,000 people in Malaysia and found that 10-year cardiovascular disease risk estimation was higher in the BMI-based model than the lipid profile-based formula for both genders [31]. Guzman-Vilca et al. used WHO CVD risk to measure 10-year CVD risk estimation and found that the agreement was substantial in men and moderate in women [30].

The results showed that two models had a good agreement among people under the age of 60 and people aged 60 years and over; however, there was a better agreement among people who were in low and high-risk groups (almost perfect and substantial). The mean risk scores of the office-based model were statistically higher than the laboratory-based model in people aged 60 years and over. Another study showed moderate agreement between laboratory-based and office-based Framingham risk score models in young and old women, but the agreement was slight in younger men and fair in older men [17]. Guzman-Vilca et al. examined the 30–59 years age group by using WHO CVD risk and showed moderate agreement in the 40–49 years age group and substantial agreement in the 50–59 years age group [30]. Jahangiry et al. examined people over the age of 40 years in the Fasa cohort study and found there was a substantial agreement between laboratory-based and office-based Globorisk in men and a moderate agreement in women [24]. Dehghan et al. showed that in the Fasa cohort study, there was a good agreement between WHO laboratory-based and non-laboratory-based models [32]. Mettananda et al. also reported substantial agreement between laboratory-based and office-based models Framingham risk scores and almost perfect agreement between laboratory-based and office-based WHO CVD risk models [33]. Although Wekesah et al. showed moderate agreement between the laboratory-based and office-based models, they suggested that office-based models should be substituted for laboratory-based models in low-middle-income countries (LMICs) with limited laboratory resources and facilities [28].

There was a good agreement between the two models in both smokers and non-smokers in all risk categories. It was shown that in the low-risk group, non-smokers were classified in the office-based model almost the same as the laboratory-based model. Although means of the risk scores in two models were different, but in smokers and non-smokers, concordance between two models was good, especially in low- and high-risk groups. One study showed that there was a moderate agreement between laboratory-based and office-based models in non-smokers and substantial agreement in smokers [30].

This study found in low- and high-risk groups, there was a suitable agreement between the two models in the diabetics vs. non-diabetics category. The agreement in low and high-risk groups was better than moderate group. Guzman-Vilca et al. used the WHO CVD risk score and showed that the agreement between laboratory-based and office-based models was substantial in diabetics and fair in non-diabetics [30].

There was a substantial agreement between the two models in BMI categories, namely normal and overweight and obesity in all risk groups. However, concordance in low-risk group was almost perfect and in moderate- and in high-risk group was substantial. Compared with the laboratory-based model, more people with overweight and obesity were in the high-risk group in the office-based model. The mean office-based model risk score was statistically higher than that of the laboratory-based model in people with overweight and obesity, but the mean laboratory-based model risk score and office-based model was not different in people with normal BMI. Guzman-Vilca et al. showed that the agreement of laboratory-based and office-based WHO CVD risk was different when stratified by BMI category; there was a substantial agreement in the normal weight and obesity categories and moderate agreement in the overweight category [30].

In general, although in people aged 60 years and over, women, non-smokers, smokers, hypertensive patients, diabetics, and people with overweight and obesity there was a significant difference between mean score of two models but this difference is very low and not clinically important.

Gaziano examined the total CVD risk scores in 9 countries and found that there was a very strong correlation between laboratory-based and office-based risk scores by sex category [34]. Jahangiry et al. observed a very strong and direct correlation between laboratory-based and office-based Globorisk [24], while Rezaei et al. observed a very strong positive correlation between WHO laboratory-based and office-based [29], and Niyibizi et al. reported a very strong positive correlation between BMI-based and lipid-based models [35]. As can be observed in scatter plots, the results obtained from kappa coefficients and correlation coefficients indicated that there was a good agreement between laboratory-based and office-based Framingham CVD risk scores in the total population and all subgroups, namely age, sex, BMI, hypertension, smoking status and diabetes status. The results of laboratory-based and office-based Framingham models were so similar that the models can be used interchangeably.

It should be noted there are some reasons for the substitution of office-based models for laboratory-based-model. First, the laboratory-based models include measurements of HDL and total cholesterol levels to determine the 10-year risk of CVDs, so it requires laboratory facilities and trained personnel; however, since office-based models don’t need to include the above-mentioned clinical measurements, they are more convenient, accessible and cost-effective. Second, obesity, which is measured by BMI is an important determinant of total cholesterol [36], which again is an independent CVD risk factor [37]. Obesity can be easily measured by the office-based model in LMICs with limited laboratory resources, facilities, and trained staff and can therefore help detect people at high-risk for CVDs.

Since most deaths are due to CVDs in low and middle-income countries [38], CVD risk prediction is of great importance to identify persons at risk early and initiate early preventive treatments and interventions. Unfortunately, laboratory-based models may be difficult to use in countries with a lack of resources and laboratory facilities. To overcome this problem, office-based models could replace laboratory-based risk prediction models.

### Study strengths and limitations

Strengths of the current study include the large sample size in a representative population sample, and use of validated tools to measure risk factors. A limitation of the study is that it had a cross-sectional study design and the Framingham Risk Score was derived in a white American population and has not been validated in this population previously. It can be noted because Iran does not have a specific risk prediction tool, this study used FRS. Further studies in this population with 10-year follow-up is therefore warranted. Also, the validity of non-laboratory-based and laboratory-based models should be determined in this population.

## Conclusion

In the laboratory-based and office-based model, about 27% of the population was in the moderate- and high-risk groups combined. The current study showed that there was a good agreement and very strong positive correlations between office-based and laboratory-based Framingham Risk Score models, which persisted across various strata of the population. For screening programs, office-based models may be more appropriate than laboratory-based models in LMICs with limited laboratory facilities and personnel. Longitudinal studies in this population are warranted. Further longitudinal studies are needed to evaluate the validity of the office-based model in this population. For moderate- and high-risk group, interventions for healthy lifestyle and timely treatment are needed.

### Supplementary Information

**Additional file 1: Table S1.** DBP diastolic blood pressure, SBP systolic blood pressure, *HDL* high-density lipoprotein, *LDL* low-density lipoprotein, *Chol* cholesterol, *TG* triglyceride, *BMI* body mass index, *SD* standard deviation.

## Data Availability

The datasets used during the current study are available from the corresponding author upon reasonable request.
