# Supplementary material for: A comparison of laboratory-based and office-based Framingham risk scores to predict 10-year risk of cardiovascular diseases: a population-based study
Source: J Transl Med. 2023 Oct 3;21:687. doi: 10.1186/s12967-023-04568-8 (PMC10546649; doi:10.1186/s12967-023-04568-8)
Supplement: Supplementary file 1 — Additional file 1: Table S1. DBP diastolic blood pressure, SBP systolic blood pressure, HDL high-density lipoprotein, LDL low-density lipoprotein, Chol cholesterol, TG triglyceride, BMI body mass index, SD standard deviation. [file 12967_2023_4568_MOESM1_ESM.docx]

**Table S1** Demographic characteristics and prevalence of Framingham risk score factors

| **Variables** | **Total**  **(n=8944)** | **Males (n=4132)** | **Females (n=4812)** | **p-value** |
| --- | --- | --- | --- | --- |
|  | **N (%)** | **N (%)** | **N (%)** |  |
| **Age** (years), **(**Mean ± SD) | 47.72±9.25 | 47.86±9.37 | 47.59±9.14 | 0.17 |
| **Education level** |  |  |  |  |
| Illiterate | 3845 (42.99) | 1350 (32.67) | 2495 (51.85) | <0.001 |
| ≤diploma | 4875 (54.51) | 2625 (63.53) | 2250 (46.76) |  |
| >diploma | 224 (2.50) | 157 (3.80) | 67 (1.39) |  |
| **Marital status** |  |  |  |  |
| Married | 7985 (89.28) | 4021 (97.31) | 3964 (82.38) | <0.001 |
| Others | 959 (10.72) | 111 (2.69) | 848 (17.62) |  |
| **Smoking (now)** |  |  |  |  |
| No | 7184(80.32) | 2463(59.61) | 4721(98.11) | <0.001 |
| Yes | 1760(19.68) | 1669(40.39) | 91(1.89) |  |
| **Hypertension** |  |  |  |  |
| No | 7558(84.50) | 3790(91.72) | 3768(78.30) | <0.001 |
| Yes | 1386(15.50) | 342(8.28) | 1044(21.70) |  |
| **Diabetes** |  |  |  |  |
| No | 7984(89.27) | 3854(93.27) | 4130(85.83) | <0.001 |
| Yes | 960(10.73) | 278(6.73) | 682(14.17) |  |
| **DBP (**Mean mmHg ± SD) | 74.30±11.77 | 74.10±11.63 | 74.48±11.89 | 0.12 |
| **SBP** (Mean mmHg ± SD) | 110.48±17.84 | 109.83±16.99 | 111.04±18.53 | 0.001 |
| **HDL** (Mean mg/dl ± SD) | 50.97±15.96 | 47.10 ±14.22 | 54.30±16.62 | <0.001 |
| **LDL** (Mean mg/dl ± SD) | 108.34±32.15 | 105.01±30.72 | 111.21±33.07 | <0.001 |
| **Chol** (Mean mg/dl ± SD) | 185.57±38.31 | 179.41±36.34 | 190.86±39.16 | <0.001 |
| **TG** (Mean mg/dl ± SD) | 130.98±82.31 | 136.09±90.97 | 126.60±73.80 | <0.001 |
| **BMI** (kg/m^2^), (Mean ± SD) | 25.55±4.81 | 24.13±4.42 | 26.77±4.80 | <0.001 |
| **BMI category** |  |  |  |  |
| Normal | 4237(47.37) | 2450(59.29) | 1787(37.14) | <0.001 |
| Overweight and obesity | 4707 (52.63) | 1682 (40.71) | 3025 (62.86) |  |

*DBP* diastolic blood pressure, *SBP* systolic blood pressure, *HDL* high-density lipoprotein, *LDL* low-density lipoprotein, *Chol* cholesterol, *TG* triglyceride, *BMI* body mass index, *SD* standard deviation
